# CaMKII inhibition reduces arrhythmogenic Ca^2+^ events in subendocardial cryoinjured rat living myocardial slices

**DOI:** 10.1085/jgp.202012737

**Published:** 2021-05-06

**Authors:** Eef Dries, Ifigeneia Bardi, Raquel Nunez-Toldra, Bram Meijlink, Cesare M. Terracciano

**Affiliations:** 1 National Heart and Lung Institute, Imperial College London, London, UK; 2 Lab of Experimental Cardiology, Department of Cardiovascular Sciences, University of Leuven, Leuven, Belgium

## Abstract

Spontaneous Ca^2+^ release (SCR) can cause triggered activity and initiate arrhythmias. Intrinsic transmural heterogeneities in Ca^2+^ handling and their propensity to disease remodeling may differentially modulate SCR throughout the left ventricular (LV) wall and cause transmural differences in arrhythmia susceptibility. Here, we aimed to dissect the effect of cardiac injury on SCR in different regions in the intact LV myocardium using cryoinjury on rat living myocardial slices (LMS). We studied SCR under proarrhythmic conditions using a fluorescent Ca^2+^ indicator and high-resolution imaging in LMS from the subendocardium (ENDO) and subepicardium (EPI). Cryoinjury caused structural remodeling, with loss in T-tubule density and an increased time of Ca^2+^ transients to peak after injury. In ENDO LMS, the Ca^2+^ transient amplitude and decay phase were reduced, while these were not affected in EPI LMS after cryoinjury. The frequency of spontaneous whole-slice contractions increased in ENDO LMS without affecting EPI LMS after injury. Cryoinjury caused an increase in foci that generates SCR in both ENDO and EPI LMS. In ENDO LMS, SCRs were more closely distributed and had reduced latencies after cryoinjury, whereas this was not affected in EPI LMS. Inhibition of CaMKII reduced the number, distribution, and latencies of SCR, as well as whole-slice contractions in ENDO LMS, but not in EPI LMS after cryoinjury. Furthermore, CaMKII inhibition did not affect the excitation–contraction coupling in cryoinjured ENDO or EPI LMS. In conclusion, we demonstrate increased arrhythmogenic susceptibility in the injured ENDO. Our findings show involvement of CaMKII and highlight the need for region-specific targeting in cardiac therapies.

## Teaser

Dries et al. show that injured myocardial slices from the subendocardium are more susceptible to spontaneous Ca^2+^ release events and whole-slice contractions than those from the subepicardium, and that this is reduced by CaMKII inhibition.

## Introduction

Dysfunctional Ca^2+^ release, such as spontaneous Ca^2+^ releases (SCRs) from the SR, play an important role in the onset of lethal ventricular arrhythmias (Pogwizd and Bers, 2004). Numerous studies using single cardiac myocytes have previously demonstrated that diastolic SR Ca^2+^ leak via RYRs can lead to propagating Ca^2+^ waves—SCR events—that generate inward sodium calcium exchange (NCX) currents. These inward currents can induce delayed afterdepolarizations (DADs) that, when of sufficient magnitude, trigger an action potential (AP) in the myocyte (Fedida et al., 1987; Kass et al., 1978; Marban et al., 1986; Pogwizd and Bers, 2004). Spontaneous Ca^2+^-induced triggered events have been generally accepted to set the stage for arrhythmia initiation; however, translating single-cell observations to the whole heart setting may be more complex, where multicellular and heterocellular connections can modulate these signaling pathways (Houser, 2000; Myles et al., 2012; Xie et al., 2010).

Advances in tissue and whole-heart models and techniques have offered a better platform with which to study the link between dysfunctional Ca^2+^ handling and the initiation of ventricular arrhythmias in a multicellular environment (Di Diego and Antzelevitch, 1994; Jaimes et al., 2016; Lee et al., 2012). Optical mapping studies with Ca^2+^ and voltage dyes in whole-heart and tissue wedge preparations successfully addressed the limitations encountered in single-cell studies (Kang et al., 2016; Lee et al., 2012; Wang et al., 2015). Initial studies in this area were limited to low-resolution mapping, whereas emerging new techniques with high spatial and temporal resolution allow measurement of individual cardiac myocyte behavior in a syncytium. These new imaging techniques were elegantly used by the group of Wasserstrom and Bers, but their observations were limited to the epicardial surface (Aistrup et al., 2006; Lang et al., 2017; Wasserstrom et al., 2010).

Intrinsic electrical transmural heterogeneity is a well-known feature of the left ventricle (LV) in healthy animals and humans (Antzelevitch et al., 1991; Boukens et al., 2015; Stankovicova et al., 2000). Experiments on single cells and wedge preparations from the LV wall have shown that the AP duration (APD) is longer in subendocardium (ENDO) myocytes versus subepicardium (EPI). This heterogeneity is attributed to differences in ion channel expression—I_to_ and I_CaL_—and accounts for activation delays and synchronized ventricular repolarization in the healthy myocardium (Bányász et al., 2003; Himmel et al., 1999; Litovsky and Antzelevitch, 1988). Furthermore, intrinsic transmural heterogeneity in mechanical features (Pitoulis et al., 2020), as well as excitation–contraction coupling (ECC) and Ca^2+^ handling (Bondarenko and Rasmusson, 2010; Dilly et al., 2006), has been shown in healthy LV myocardium. Previous work by Laurita and Katra (2005) showed that the decline of the Ca^2+^ transients in canine LV was slower in cells near the ENDO versus cells from the EPI. Others observed that the latency to onset of contraction was shorter and SR Ca^2+^ content was higher in EPI cells as compared with ENDO cells in normal canine LV (Cordeiro et al., 2004); however, little is known about the transmural differences in the occurrence and properties of arrhythmogenic SCR events in the intact LV myocardium. Moreover, the intrinsic LV transmural heterogeneity is subject to remodeling with disease. In failing human hearts, heterogeneous changes in SERCA2a expression, as well as ECC and global Ca^2+^ handling, have been reported throughout the LV wall (Lou et al., 2011; Prestle et al., 1999). How disease remodeling functionally affects SCR events in different transmural regions is currently unknown.

The use of living myocardial slices (LMSs) has earlier been validated in cardiovascular research, including electrophysiological and pharmacology studies (Camelliti et al., 2011; Fischer et al., 2019; George et al., 2020; Himmel et al., 2012; Kang et al., 2016; Wang et al., 2015; Watson et al., 2019, 2017), and represents a useful tool with which to study transmural differences of cardiac function in their native environment (Pitoulis et al., 2020; Wen et al., 2018). LMSs can be prepared from the LV of a number of species and have been shown to retain structural and functional properties of the native myocardium, including tissue architecture, cell type ratio, cell–cell and mechanical coupling, and extracellular matrix (Perbellini et al., 2018; Watson et al., 2017). While earlier studies were limited to the preparation of healthy LMS, recent work has addressed underlying disease mechanisms in LMS from diseased hearts (Fischer et al., 2019; Kang et al., 2016; Watson et al., 2019); however, it remains technically challenging to prepare viable LMSs from diseased hearts with high levels of fibrosis, such as that seen during myocardial infarction. A simpler and reproducible approach is to replicate the pathological stimuli in vitro using healthy LMS.

In this study, we used the novel technique of LMS subjected to cryoinjury to mimic cardiac disease. This in vitro model was then used to investigate single SCR events in different regions of the LV wall by high-resolution imaging of individual cells in a syncytium. The goal of this study was to investigate (1) the effect of cardiac injury on the occurrence and kinetics of SCR events in different transmural regions of the myocardium, and (2) the effect of Ca^2+^-modulating drugs on these potentially arrhythmogenic Ca^2+^ events.

## Materials and methods

### Ethical approval

All animal experiments in this study were performed according to institutional and national regulations. Use of living cardiac tissue was approved by Imperial College London and the procedures described here were completed under license by the UK Home Office, following the UK Animals (Scientific Procedures) Act 1986. Animals were housed and treated according to the Guide for the Care and Use of Laboratory Animals (National Institutes of Health) and the European Directive 2010/63/EU. Animals were euthanized and hearts were collected to prepare LMS. In brief, male Sprague-Dawley rats (±300 g) were sedated with isoflurane (4% isoflurane mixed with 4 liters/min oxygen) and sacrificed using cervical dislocation and dissection of the carotid arteries.

### Preparation of LMSs

**Figure S1.**
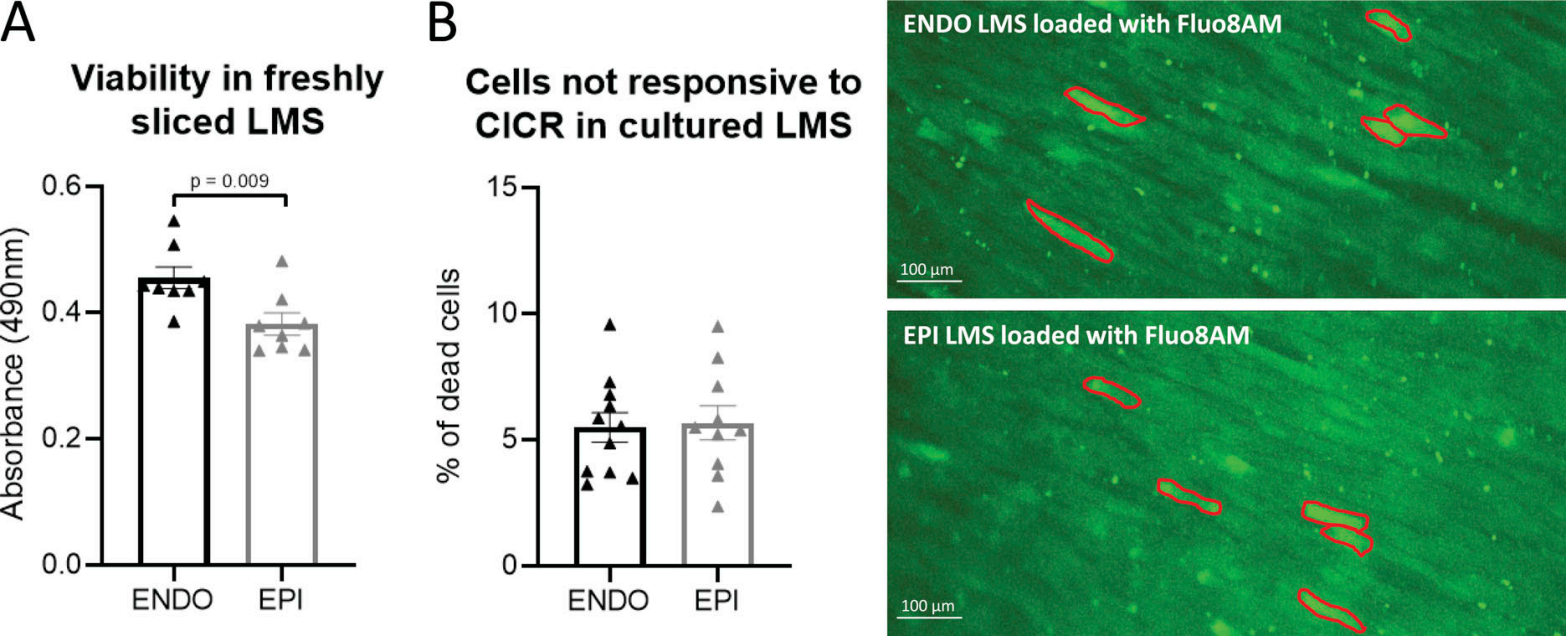
**Viability in freshly sliced LMS and after 24 h of culture.** **(A)** The viability in freshly sliced LMS was tested using a colorimetric MTS assay to measure cellular metabolic activity in ENDO and EPI LMS. Viable and metabolic active cells reduced yellow MTS to purple formazan. The resulting colored solution was quantified by measuring absorbance at 490 nm. The more absorbance detected at 490 nm, the greater the number of viable and metabolic active cells. Freshly sliced ENDO LMS showed a higher viability compared with freshly sliced EPI LMS (*n*_endo_ = 8, *n*_epi_ = 8 from four rats; data are compared using Student’s *t* test). **(B)** The viability of 24-h cultured LMS was assessed based on their responsiveness to calcium-induced calcium release (CICR). After loading LMS with the fluorescent Ca^2+^ indicator Fluo-8AM, electrical stimulation will induce CICR (visible as a change in fluorescence) in viable cells. Cells not responsive to CICR were assumed to be dead cells. These cells mostly showed up with a very bright fluorescent signal (contoured in red in B), in accordance with Ca^2+^ overload and cell death. On average, 5% of cells on the superior layer of LMS were not responsive to electrical stimulation, both in cultured ENDO and EPI LMS (*n*_endo_ = 11, *n*_epi_ = 10 from 10 rats; data are compared using Student’s *t* test).

Preparation of LMSs was done according to a protocol previously established by our laboratory (Watson et al., 2017). The rat heart was immediately removed and placed in warm (37°C) and later ice-cold (4°C), heparinized (2.4 IU/ml), normal Tyrode’s solution (in mmol/liter: 140 NaCl, 6 KCl, 1 MgCl_2_, 1 CaCl_2_, 10 HEPES, and 10 glucose, pH 7.4 with NaOH) containing 30 mM 2,3-butanedione monoxime (BDM) to remove all excessive blood from the heart. The LV was isolated, opened flat, and mounted onto an agarose-coated specimen holder using tissue glue (HistoAcryl; Braun) with the epicardial surface facing down. A high-precision vibrating microtome (7000 smz-2; Campden Instruments) was used to prepare 300-µm thin LMSs with a vibrating frequency of 80 Hz, amplitude of 2 mm, and advance speed of 0.03 mm/s. The orientation of the tissue block ensured that the ceramic blade cut in parallel to the fibers’ orientation of the LV to minimize the tissue damage (Fig. S1). Throughout the slicing process, the tissue was constantly submerged in ice-cold (4°C) oxygenated Tyrode’s solution containing 30 mM BDM. Different regions of the LV wall were studied as follow: subendocardial slices were obtained ≤600 µm from the endocardial surface, and subepicardial slices were obtained ≤600 µm from the epicardial surface. After preparation, LMSs were used immediately for culture experiments and randomly assigned to control or cryoinjury groups. Cryoinjury was performed using a 3-mm cylindrical rod made out stainless steel that was cooled down on dry ice (−78.5°C). The rod was placed carefully on the tissue for 2–3 s before the culture time and immediately removed, resulting in an injury (Video 1; Strungs et al., 2013).

**Video 1.** **Induction of cryoinjury on a freshly cut LMS.** Cryoinjury was performed using a 3-mm cylindrical rod made out stainless steel that was cooled down on dry ice (−78.5°C). The rod was placed carefully on the tissue for 2–3 s before the culture time and immediately removed, resulting in an injury. Playback at 24 frames/s.

### Mechanical loading of myocardial slices

**Figure 1.**
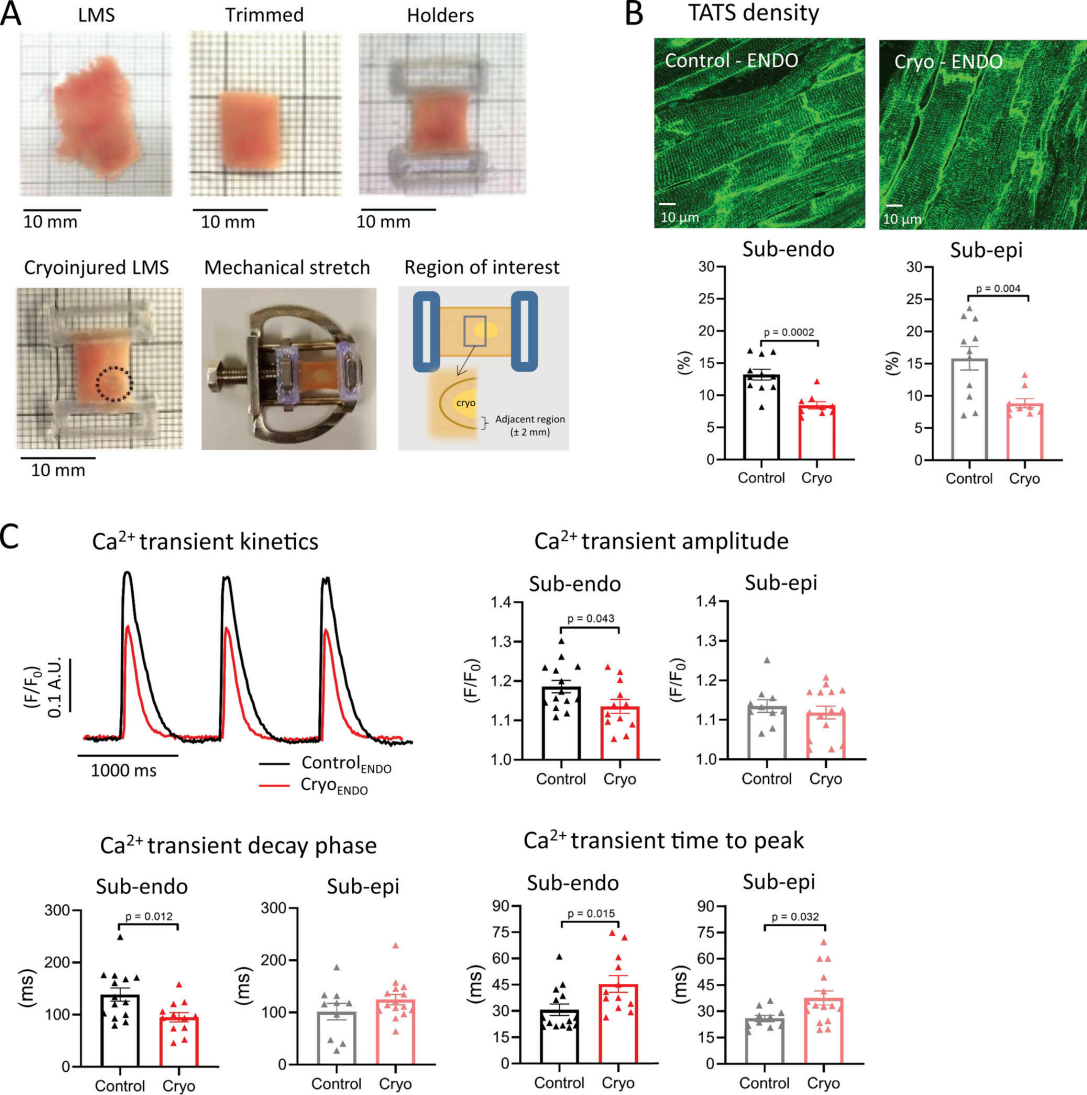
**Cryoinjury of LMS induces local remodeling in the region adjacent to the injury.** **(A)** Examples of LMS during different stages of preparation. A freshly cut LMS is trimmed according to the fiber orientation. The trimmed LMS is attached to holders using tissue glue. After attachment to the holders, cryoinjury was induced using a 3-mm ice-cold cylindrical rod. Lastly, the LMS is positioned onto a stretcher to allow mechanical loading. The region adjacent to the cryoinjury (<2 mm) was studied to investigate SCR events in regions of the LV wall. **(B)** Examples and mean values of TATS in LMS from the ENDO (*n*_control_ = 11 from 11 rats; *n*_cryoinjury_ = 9 from 9 rats; comparison with Student’s *t* test) and from the EPI (*n*_control_ = 11 from 10 rats; *n*_cryoinjury_ = 9 from 8 rats; comparison with Student’s *t* test). **(C)** Examples and mean values of kinetics of Ca^2+^ transients at 1-Hz pacing in LMS from the ENDO (*n*_control_ = 14 from 12 rats; *n*_cryoinjury_ = 12 from 12 rats; comparison with Student’s *t* test) and from the EPI (*n*_control_ = 10 from 9 rats; *n*_cryoinjury_ = 15 from 15 rats; comparison with Student’s *t* test).

After slicing, LMS fiber orientation was assessed under a stereomicroscope and a rectangular region (width 7 mm × length 8 mm) with homogenous fiber alignment was trimmed using a razor blade. The size of the LMS was kept constant to minimize variability between samples (Fig. 1 A). Custom-made holders made of biocompatible polyethylene terephthalate (Taulman3D T-glase) were attached to either side of the LMS using surgical glue (HistoAcryl; Braun) and placed perpendicular to the fibers’ orientation, which allowed the tissue to stretch along the fibers (Fig. 1 A). The resting length and width of the LMS were measured using calipers and then stretched at sarcomere length of 2.2 µm, as previously reported by our laboratory (Watson et al., 2019). Sterile, custom-made, stainless-steel stretchers were used to apply fixed mechanical load to the tissue (Fig. 1 A; Watson et al., 2019).

### Culture of LMSs

Prior to culturing, all tools and instrumentation were autoclaved and all handling of LMSs was performed in a sterile laminar flow cabinet. LMSs were cultured for 24 h at 37°C in culture media (medium-199 + 0.1% insulin-transferrin-selenium + 3% penicillin-streptomycin; all Sigma-Aldrich) with electrical stimulation using carbon electrodes at a pulse frequency of 1 Hz and pulse width of 10 ms at 15 V. LMSs were constantly oxygenated (95% O_2_ and 5% CO_2_). Constant recirculation with a peristaltic pump was used to ensure circulation of oxygen into the culture medium. Control and cryoinjured groups were cultured in separate culture chambers to avoid interference of autocrine modulation.

### Calcium imaging

After culture, slices were carefully removed from the culture chamber and incubated for 15 min at 37°C with the calcium indicator Fluo-8AM (10 µM; ab142773; Abcam) in normal Tyrode solution (substitute 6 mM KCl with 4.5 mM KCl) with 0.1% pluronic F-127 (Thermo Fisher Scientific). Incubation of the slice was done on the custom-made stretcher to maintain specific sarcomere length as that during culture. After incubation, LMSs were transferred to an upright microscope (Nikon Eclipse FN1) equipped with a Hamamatsu Orca Flash 4.0 LT CMOS camera and constantly perfused with oxygenated normal Tyrode solution at 37°C (substitute 6 mM KCl with 4.5 mM KCl) containing 10 mM BDM and 10 µM blebbistatin to reduce motion artifacts. Fluo-8AM was excited using an LED light source at 470 nm (Cairn OptoLED) and emission signals were collected using a 495-nm long-pass dichroic mirror with a 10× objective (numerical aperture 0.3; Plan Fluor). LMSs were first field stimulated at 1 Hz (15 V, 20-ms bipolar pulse) to induce calcium transients. Calcium transients were recorded in a 2048 × 512-pixel image at 154 frames/s during a 5-s period using HCImage Live software (Hamamatsu). Next, LMSs were conditioned with a pro-arrhythmic pacing protocol for 2 min: field stimulation at 4–6 Hz (15 V, 20-ms bipolar pulse) and isoproterenol (1 µM). After 2 min, pro-arrhythmic pacing was stopped and SCR events were detected in a 2048 × 512-pixel image at 100 frames/s during a 20-s period using HCImage Live software (Videos 2 and 3). Calcium imaging was done in control and cryoinjured slices without and with the specific calcium/calmodulin-dependent kinase II (CaMKII) blocker, myristoylated autocamtide-2–related inhibitory peptide (AIP; 10 µM; incubation for 2 h at culture conditions as described above).

**Video 2.** **SCR events after pro-arrhythmic stimulation in control LMS.** LMSs were conditioned with pro-arrhythmic pacing and SCR events were recorded after stimulation at 100 frames/s using HCImage Live software. Scale bar, 100 µm. Playback at 24 frames/s.

**Video 3.** **SCR events after pro-arrhythmic stimulation in cryoinjured LMS.** LMSs were conditioned with pro-arrhythmic pacing and SCR events were recorded after stimulation at 100 frames/s using HCImage Live software. Cryoinjury is shown on the left of the video below the scale bar. Scale bar, 100 µm. Playback at 24 frames/s.

### Force measurements

LMSs’ active force was assessed using a force transducer (Harvard Apparatus). Cultured slices were carefully removed from the culture chamber and from custom-made stretchers. Slices were then attached to the force transducers using the attached polyethylene terephthalate rings. Slices were constantly perfused with 37°C oxygenated normal Tyrode’s solution (substitute 6 mM KCl with 4.5 mM KCl, without BDM and blebbistatin) and field stimulated at 1 Hz using 10–30 V. Slices were stepwise stretched until maximum isometric contraction was reached. Data were recorded using AxoScope software and peak amplitude was analyzed using Clampfit (Molecular Devices).

### LMS viability

Tissue viability was assessed on freshly prepared LMS using a colorimetric method with the CellTiter 96 AQueous One Solution Cell Proliferation Assay (3-(4,5-dimethylthiazol-2-yl)-5-(3-carboxymethoxyphenyl)-2-(4-sulfophenyl)-2H-tetrazolium [MTS]; Promega). A tissue puncher (7 mm^2^ area) was used to create comparable size and volume of LMSs in ENDO and EPI. LMSs were then blot dried on tissue paper and placed into a 96-well plate (VWR) filled with culture media. The CellTiter solution was then added as per manufacturer’s instructions. The tissue was incubated in a humidified and oxygenated incubator at 37°C for 20 min. After incubation, media was mixed and transferred into new wells so that absorbance could be measured at 490 nm and analyzed using SoftMax Pro 6.4.2 (Molecular Devices).

### Immunostaining and confocal imaging

After culture, LMSs were washed in PBS and then fixed in 4% paraformaldehyde solution for 30 min at room temperature. Slices were permeabilized in 1% Triton X-100 for 1 h at room temperature. Slices were then blocked using blocking buffer (10% FBS, 5% BSA, and 10% horse serum in PBS) for 2 h at room temperature. LMSs were incubated with the primary antibody overnight at 4°C in blocking buffer and then washed three times for 30 min with PBS. LMSs were next incubated with secondary antibody in blocking buffer for 3 h at room temperature and then washed three times for 30 min with PBS. LMSs were then stored in PBS at 4°C. Imaging of immunolabeled LMS was done with a confocal microscope (Zeiss LSM780) using a 63× oil objective with numerical aperture of 1.4. Primary antibodies were as follows: mouse IgG anti–caveolin-3 (1/500; 610421; BD Biosciences), rabbit IgG anti-connexin43 (Cx43; 1/2,000; C6291; Sigma-Aldrich), mouse IgG anti-RYR (1/200; MA3-925; Thermo Fisher Scientific), and rabbit IgG anti-phosphor2814-RYR (1/200; A010-31AP; Badrilla); and secondary antibodies were as follows: goat anti-mouse IgG Alexa568 (1/2,000; Life Technologies) and goat anti-rabbit IgG Alexa488 (1/2,000; Life Technologies).

### Image analysis

Ca^2+^ transients were analyzed after background subtraction in ImageJ. Ca^2+^ transient amplitude, time to peak, and decay phase were measured. Ca^2+^ transient amplitude was reported as the peak fluorescence (F) normalized to baseline values (F_0_). Analysis of SCR events was performed after background subtraction and using custom-made macros in ImageJ. For each LMS, the origins of SCR events were manually identified at rest, creating a mask of SCR foci. From these masks the number of SCR foci and distances to the nearest SCR foci were calculated using the nearest neighbor distance plugin in ImageJ (Haeri and Haeri, 2015). The number of foci was quantified as the number of spots with SCR per tissue area and reported as the number of events per square millimeter. The first latency timing was quantified from the time after the last stimulation pulse to the time of first SCR. Speed of SCRs, distances of SCR propagation, and the angles of SCR propagation were analyzed.

Sarcolemma and transverse and axial tubules (TATS) were visualized by immunostaining using the primary antibody anti-mouse IgG caveolin-3 and secondary antibody goat anti-mouse IgG Alexa568. Confocal images were collected and analyzed using ImageJ. TATS signals within the cell margins were identified against the background by thresholding. The sarcolemmal membrane was subtracted from thresholded images. Thresholded images were further processed to skeletonize TATS signals. TATS density was quantified as the number of signal-positive pixels over all pixels within the cell margins. Cx43 signals were thresholded to identify Cx43 clusters against the background. Cx43 cluster area was then normalized to the total myocyte area. Analysis of Cx43 cluster lateralization was based on the quantification of the angle formed between the local longitudinal cell axis and the main axis of the individual connexins cluster, as previously described by Burstein et al. (2009). The intensity of phosphor2814-RYR was analyzed in RYR clusters. After background subtraction, filtering, and local thresholding, a mask of RYR clusters was generated. This mask was used to identify RYR clusters and measure the fluorescence intensity of RYR and phosphor2814-RYR. Fluorescence intensity of phosphor2814-RYR was normalized to the fluorescence intensity of its respective RYR cluster.

### Experimental design and statistics

Data acquisition was performed by multiple investigators. Investigators were blinded when doing analysis off-line by creating coded data files. Individual data are shown in dot plots, with each dot representing the mean value of one LMS with SEM bars. Datasets were tested for normality using Shapiro-Wilk testing. Normally distributed data were compared using Student’s *t* test or a two-way ANOVA with Bonferroni post-hoc testing as applicable. Data that did not pass the normality test were compared using the Mann-Whitney test as applicable. Curve fittings were done using log-normal Gaussian fitting, and geometric means and geometric SDs were compared. Data were considered significantly different when the P value was <0.05, with exact P values shown in the graphs.

### Online supplemental material

**Figure S2.**
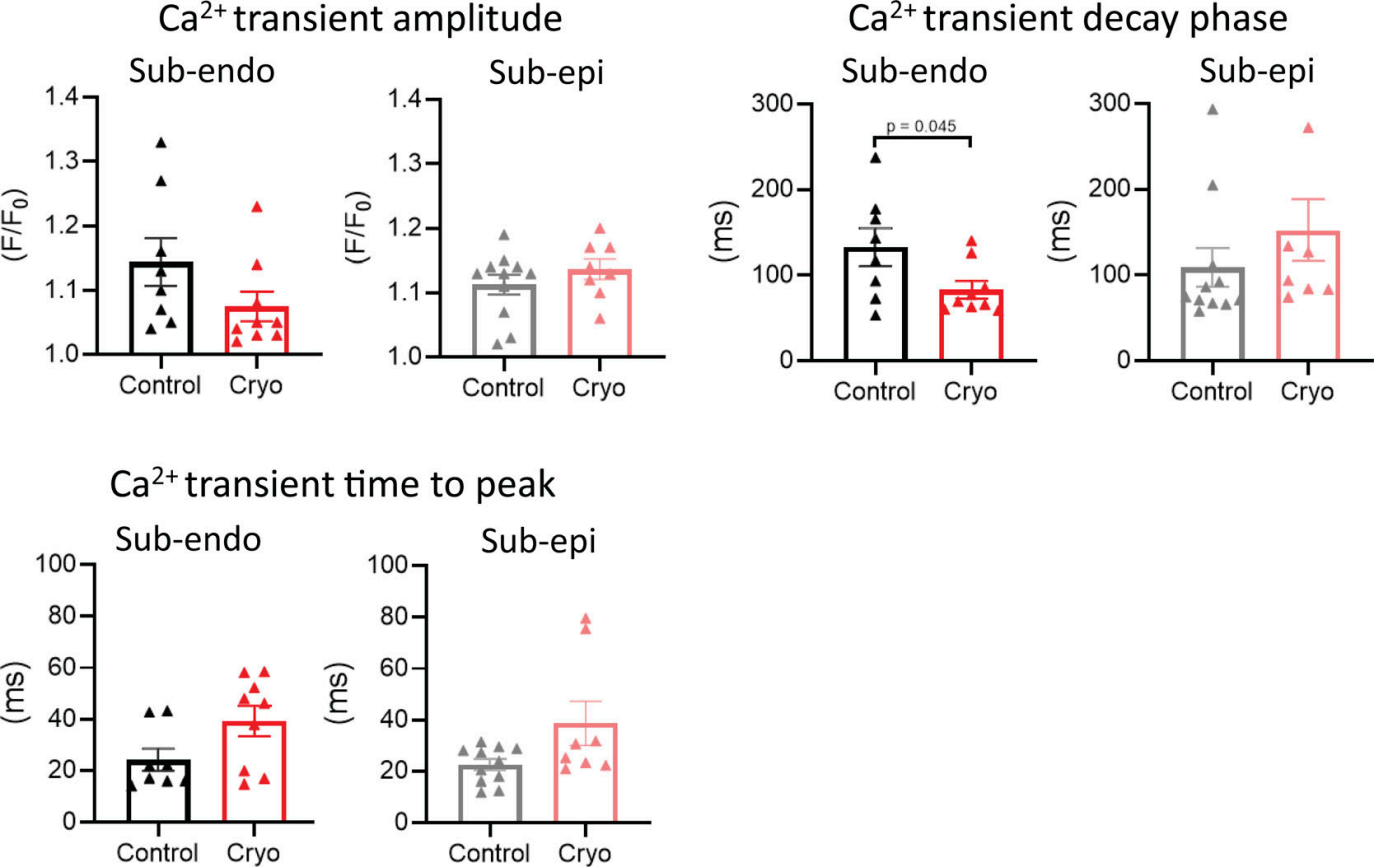
**Ca^2+^ transient kinetics during pro-arrhythmic pacing.** Ca^2+^ transient amplitude, decay phase, and time to peak during pro-arrhythmic pacing in LMS from the ENDO (*n*_control_ = 8 from 8 rats, *n*_cryo_ = 9 from 9 rats) and EPI (*n*_control_ = 11 from 10 rats, *n*_cryo_ = 8 from 8 rats; data are compared using Student’s *t* test).

**Figure S3.**
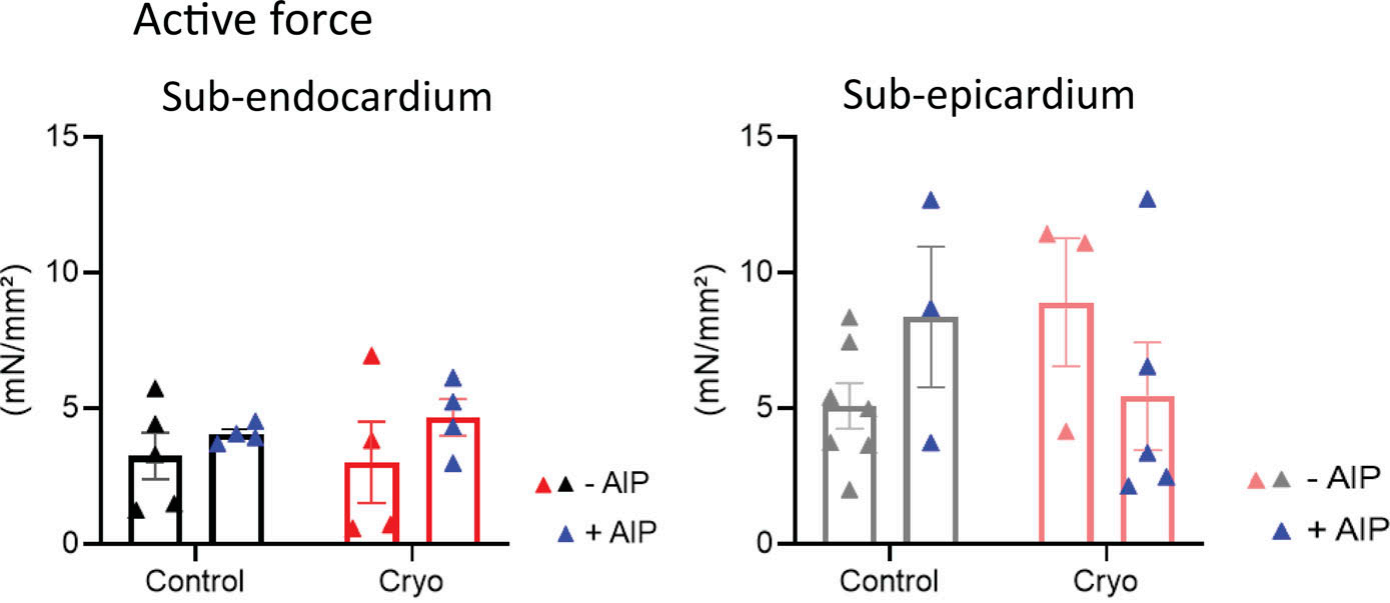
**CaMKII inhibition does not affect the development of active force.** Mean values of active force measurements during 1-Hz pacing in LMS from the ENDO in the absence (*n*_control_ = 5 from five rats; *n*_cryoinjury_ = 4 from four rats) and presence of AIP (*n*_control_ = 4 from four rats; *n*_cryoinjury_ = 4 from four rats), and from the EPI in the absence (*n*_control_ = 7 from seven rats; *n*_cryoinjury_ = 3 from three rats) and presence of AIP (*n*_control_ = 3 from three rats; *n*_cryoinjury_ = 5 from five rats; comparison with two-way ANOVA with Bonferroni post-hoc testing).

Fig. S1 shows viability measurements of ENDO and EPI LMS before and after culturing. Fig. S2 shows Ca^2+^ transient kinetics during pro-arrhythmic pacing. Fig. S3 shows active force development during 1-Hz pacing in the absence and in the presence of AIP. Video 1 demonstrates the induction of cryoinjury on LMS. Video 2 and Video 3 are recordings of SCR events after pro-arrhythmic stimulation****in control (Video 2) and cryoinjured (Video 3) LMS.

## Results

### Cryoinjury induces local remodeling adjacent to the injury in ENDO and EPI LMS

Fresh LMSs were cryoinjured as described above (±12% of total tissue area; Video 1) and stretched to mimic mechanical loading of the LV (Fig. 1 A). After 24 h of culture, SCR events were studied in the region bordering the cryoinjury (<2 mm; Fig. 1 A). This border zone is of particular interest due to its putative causal role in reentry and triggered arrhythmias (Baba et al., 2005; Dries et al., 2020; Hegyi et al., 2018). In this local region, we observed classic markers of disease remodeling. TATS density was significantly reduced in ENDO and EPI LMS after cryoinjury (Fig. 1 B). Consistent with the loss of TATS, the Ca^2+^ transient showed an increased time to peak after injury (Fig. 1 C). Moreover, in ENDO LMS, the Ca^2+^ transient amplitude and decay phase were significantly reduced, while these parameters were not affected in EPI LMS after cryoinjury (Fig. 1 C).

### Cryoinjured slices from the ENDO have a higher arrhythmogenic potential

**Figure 2.**
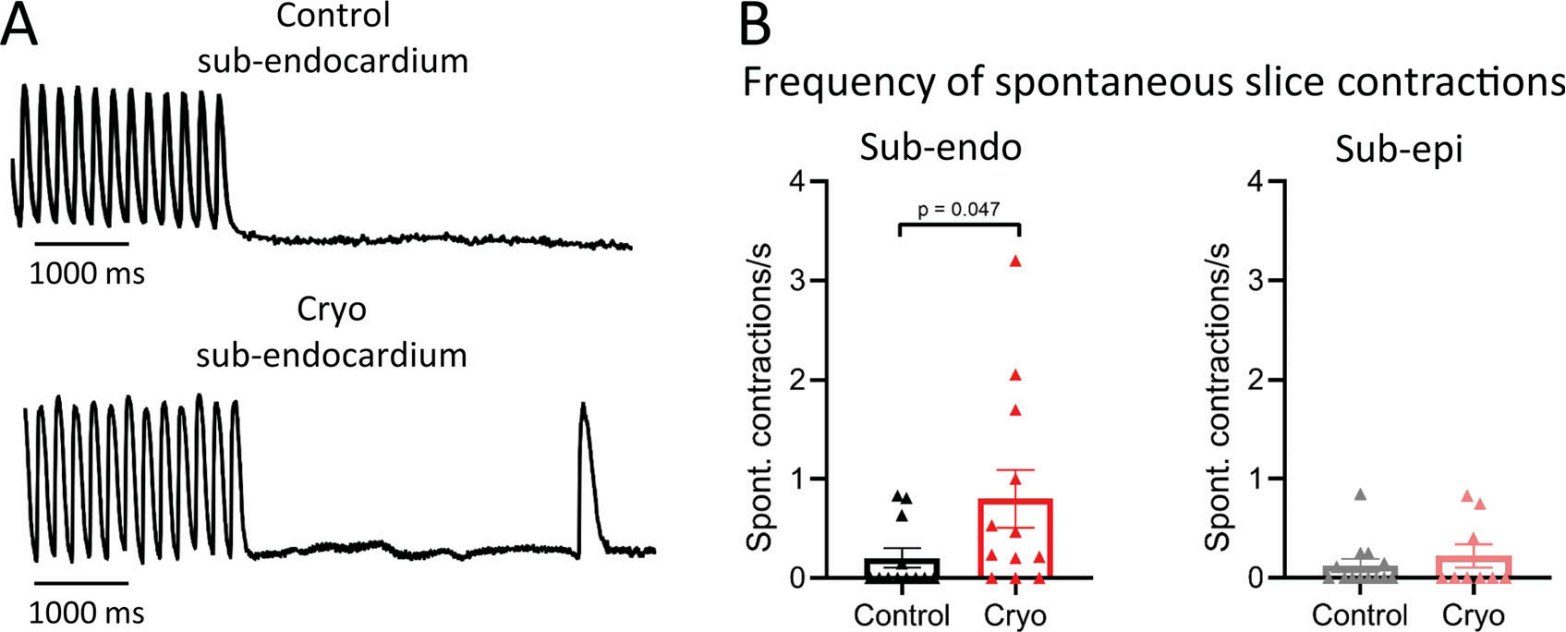
**More spontaneous whole-slice contractions in cryoinjured LMSs from the ENDO.** **(A)** Examples of pro-arrhythmic pacing and spontaneous contractions in control and cryoinjured LMSs from ENDO. **(B)** Mean values of the frequency of spontaneous contractions in LMS from the ENDO (*n*_control_ = 12 from 11 rats; *n*_cryoinjury_ = 12 from 10 rats; comparison with Mann-Whitney test) and from the EPI (*n*_control_ = 13 from 12 rats; *n*_cryoinjury_ = 9 from 9 rats; comparison with Mann-Whitney test).

We first assessed the potential of inducing arrhythmias in cryoinjured LMS by conditioning slices with a pro-arrhythmic stimulation protocol—4–6 Hz in presence of isoproterenol—followed by the abrupt termination of pacing to induce spontaneous or ectopic beats. Spontaneous beats were recorded for 15 s during rest (Fig. 2 A). After cryoinjury, the frequency of spontaneous contractions was significantly increased in ENDO LMS, while the frequency of spontaneous contractions was not affected in EPI LMS (Fig. 2 B).

### More and closely distributed SCR events in cryoinjured slices from the ENDO

**Figure 3.**
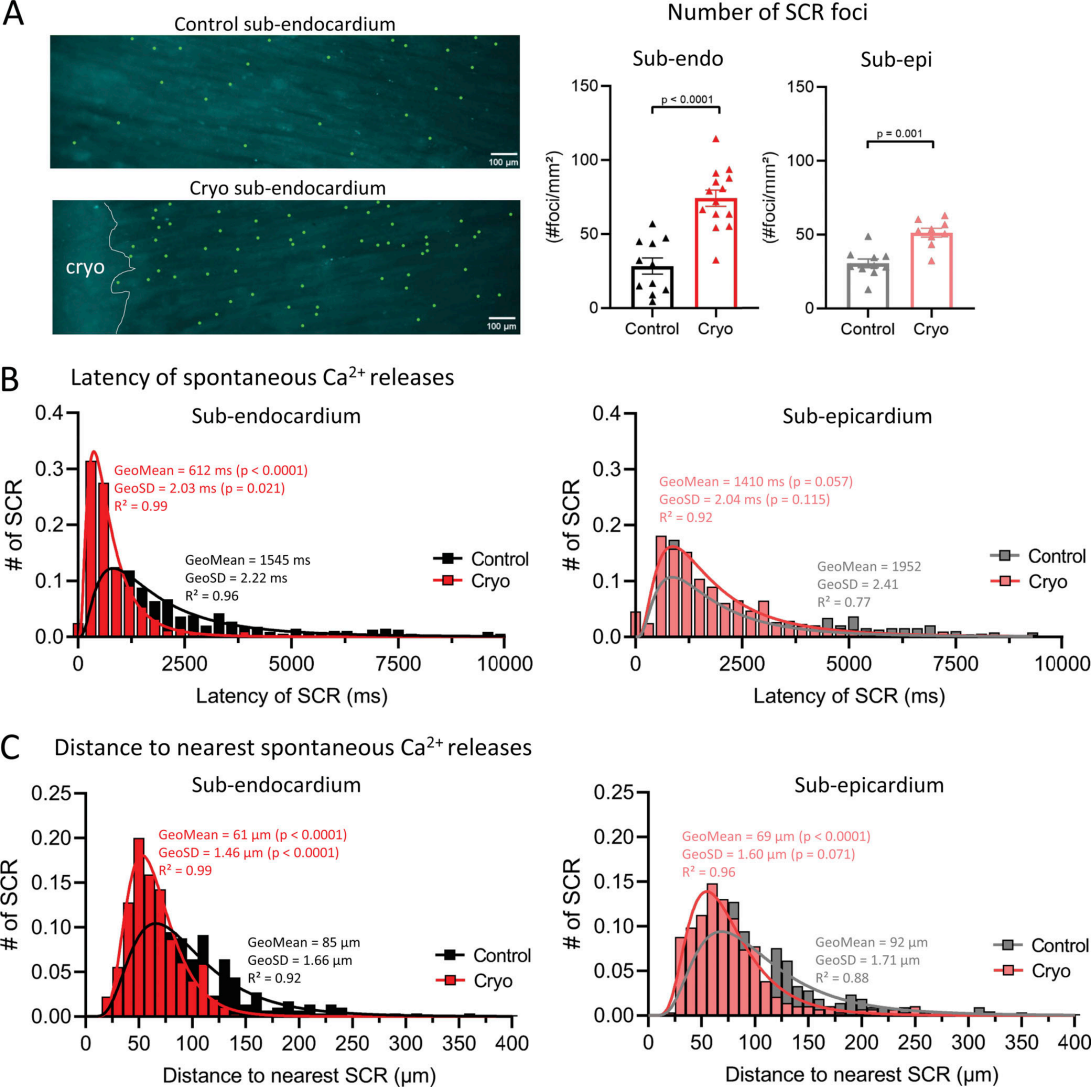
**More and closely distributed SCR events in cryoinjured ENDO slices.** **(A)** Left: Map of SCR foci in control and cryoinjured ENDO LMS after pro-arrhythmic pacing. Green dots represent single SCR foci and cryoinjury is indicated with white dotted line. Right: Mean values of the number of SCR foci in LMS from the ENDO (*n*_control_ = 11 from 11 rats; *n*_cryoinjury_ = 14 from 12 rats; comparison with Student’s *t* test) and from the EPI (*n*_control_ = 10 from 9 rats; *n*_cryoinjury_ = 9 from 8 rats; comparison with Student’s *t* test. **(B)** Distribution plots of the first latencies in LMS from the ENDO (*n*_control_ = 11 from 11 rats; *n*_cryoinjury_ = 14 from 12 rats) and from the EPI (*n*_control_ = 10 from 9 rats; *n*_cryoinjury_ = 9 from 8 rats; comparison of GeoMean and GeoSD in ENDO and EPI after lognormal curve fitting). **(C)** Distribution plots of distance to the nearest SCR foci in LMS from the ENDO (*n*_control_ = 11 from 11 rats; *n*_cryoinjury_ = 14 from 12 rats) and from the EPI (*n*_control_ = 10 from 9 rats; *n*_cryoinjury_ = 9 from 8 rats; comparison of GeoMean and GeoSD in ENDO and EPI after lognormal curve fitting).

SCR events are the precursors in triggering ectopic beats that can initiate ventricular arrhythmias. We next investigated the occurrence and kinetics of single SCR events in ENDO and EPI LMS with and without cryoinjury. LMSs were conditioned with a pro-arrhythmic pacing protocol and SCR events were recorded for 15 s during rest (Videos 2 and 3). Fig. 3 A shows an example map of foci where SCR originated (green dots) in control and cryoinjured ENDO LMSs. After cryoinjury, the number of foci that generated SCR events was significantly increased by 2.6-fold and 1.7-fold in ENDO and EPI LMS, respectively.

The likelihood of inducing triggered activity depends on the simultaneous activation of multiple SCR events in close proximity; thus, a higher number of SCR events at the same time and in close proximity can increase the likelihood of initiating triggered activity. Next, we examined the distribution of first latency timings—that is, the time between the last pacing event and when the first SCR event occurs—and the spatial distribution of SCR foci in control and injured LMSs (Fig. 3, B and C).

After cryoinjury, ENDO LMS showed a significant left shift in the first latency distribution plot compared with control ENDO LMS. Moreover, the first latencies showed a narrower distribution in cryoinjured ENDO LMS (Fig. 3 B), indicating that SCR events occur more synchronized in the injured ENDO. After cryoinjury, the distribution of first latencies was not affected in EPI LMS (Fig. 3 B).

Furthermore, the distribution of distances to the nearest SCR showed a significant left shift in ENDO LMS. This distribution was significantly narrower compared with control ENDO LMS, indicating more closely distributed SCR foci in ENDO LMS after cryoinjury (Fig. 3 C). The distribution of distances to the nearest SCR foci was also left shifted in EPI LMS after cryoinjury, but distances were equally distributed in control and cryoinjured LMSs (Fig. 3 C).

### SCR events propagate faster and further in a longitudinal direction in cryoinjured subendocardial slices

**Figure 4.**
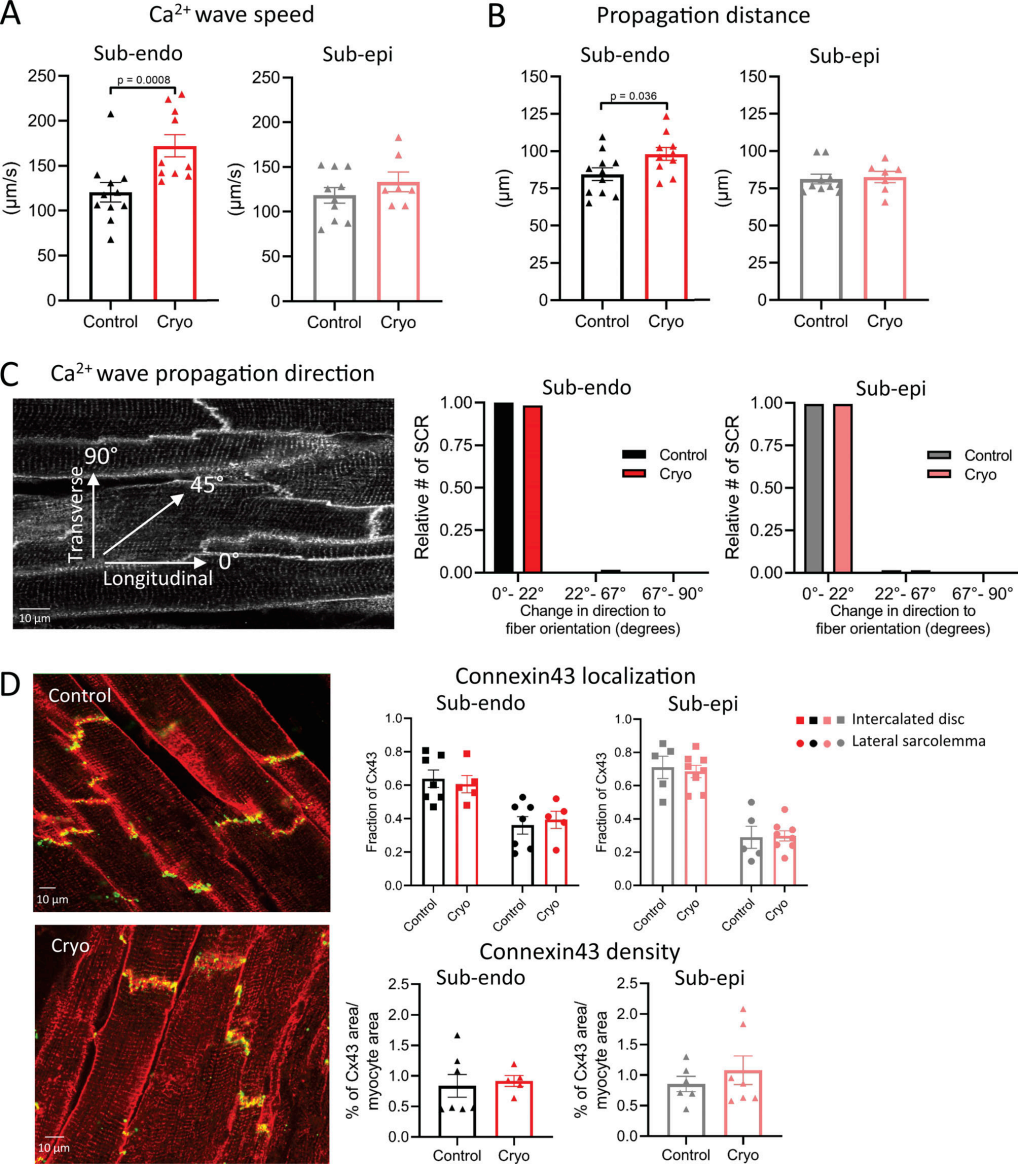
**SCR events propagate faster and further in a longitudinal direction in cryoinjured ENDO LMSs.** **(A and B)** Mean values of SCR speed (A) and distance propagation in LMS from the ENDO (B; *n*_control_ = 11 from 11 rats; *n*_cryoinjury_ = 10 from 10 rats; comparison with Student’s *t* test) and from the EPI (*n*_control_ = 10 from 9 rats; *n*_cryoinjury_ = 7 from 7 rats; comparison with Student’s *t* test). **(C)** Direction of propagation of SCR in LMS from the ENDO (*n*_control_ = 11 from 11 rats; *n*_cryoinjury_ = 10 from 10 rats) and from the EPI (*n*_control_ = 10 from 9 rats; *n*_cryoinjury_ = 7 from 7 rats; comparison with Student’s *t* test). **(D)** Mean values of Cx43 localization and Cx43 cluster density in LMS from the ENDO (*n*_control_ = 7 from six rats; *n*_cryoinjury_ = 5 from five rats) and from the EPI (*n*_control_ = 6 from five rats; *n*_cryoinjury_ = 7 from five rats; Cx43 localization was analyzed using two-way ANOVA with Bonferroni post-hoc testing; Cx43 cluster density was analyzed using Student’s *t* test).

We further examined the kinetics of SCR events in different LV regions. After cryoinjury, the propagation of SCR events was significantly faster in ENDO LMS, but was not affected in EPI LMS (Fig. 4 A). Moreover, after cryoinjury, the propagation distance of SCR events was significantly increased in ENDO LMS, but was not affected in EPI LMS (Fig. 4 B). The direction of propagation of these SCR events in both ENDO and EPI LMSs was in a predominantly longitudinal direction after cryoinjury (Fig. 4 C). Consistent with this longitudinal direction of travel, we did not find any increased Cx43 lateralization in ENDO or EPI LMS after cryoinjury (Fig. 4 D). In addition, Cx43 density was also not altered in ENDO and EPI LMS after cryoinjury (Fig. 4 D).

### CaMKII inhibition reduces the occurrence of SCR in cryoinjured subendocardial slices

**Figure 5.**
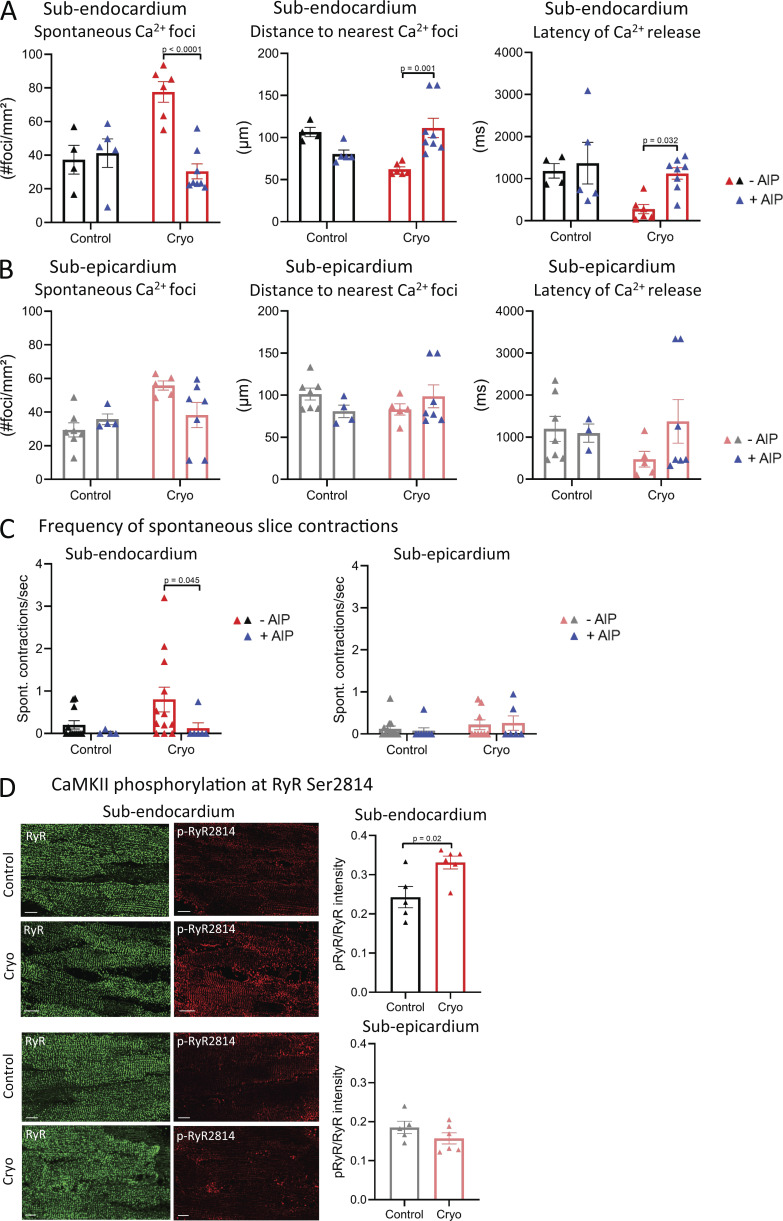
**CaMKII inhibition reduces SCR events in cryoinjured slices from the ENDO.** **(A and B)** Mean values of SCR occurrence and kinetics in LMS from the ENDO in the absence (*n*_control_ = 4 from four rats; *n*_cryo_ = 6 from six rats) and presence of AIP (*n*_control_ = 5 from five rats; *n*_cryo_ = 8 from seven rats; comparison with two-way ANOVA with Bonferroni post-hoc testing; A), and in the EPI in the absence (*n*_control_ = 7 from seven rats; *n*_cryo_ = 5 from five rats) and presence of AIP (*n*_control_ = 4 from four rats; *n*_cryo_ = 7 from six rats; comparison with two-way ANOVA with Bonferroni post-hoc testing; B). **(C)** Mean values of the frequency of spontaneous slice contractions in LMS from the ENDO in the absence (*n*_control_ = 12 from 11 rats; *n*_cryo_ = 12 from 10 rats) and presence of AIP (*n*_control_ = 4 from 4 rats; *n*_cryo_ = 6 from 6 rats), and from the EPI in the absence (*n*_control_ = 13 from 12 rats; *n*_cryo_ = 9 from 9 rats) and presence of AIP (*n*_control_ = 8 from 7 rats; *n*_cryo_ = 6 from 5 rats; comparison with Kruskal-Wallis ANOVA with Bonferroni post-hoc testing). **(D)** Mean values of p-RYR2814/RYR intensity in LMS from the ENDO (*n*_control_ = 5 from five rats; *n*_cryo_ = 6 from six rats) and EPI (*n*_control_ = 5 from five rats; *n*_cryo_ = 6 from six rats; comparison with Student’s *t* test). Scale bar, 10 μm.

Dysfunctional RYRs are responsible for SCR events (Pogwizd and Bers, 2004). An important role for increased CaMKII-mediated phosphorylation of the RYR, thereby increasing the channel’s open probability (P_o_), has been suggested in the induction of SCR events and the initiation of arrhythmias (Belevych et al., 2017; Dries et al., 2018; Fischer et al., 2014; Sag et al., 2009). Using AIP, we next assessed the impact of CaMKII inhibition on the occurrence and properties of SCR after conditioning LMS with a pro-arrhythmic protocol. In cryoinjured ENDO LMS, AIP decreased SCR foci, while increasing first latency timings and distance to nearest SCR foci (Fig. 5 A). In cryoinjured EPI LMS, AIP did not affect number of foci, first latency timings, or distance to nearest foci (Fig. 5 B). Moreover, AIP significantly reduced the frequency of spontaneous whole-slice contractions in ENDO LMS, but did not affect EPI LMS after cryoinjury (Fig. 5 C). These data may indeed suggest a role for increased CaMKII-mediated modulation of RYRs in ENDO LMS after cryoinjury. To confirm this, we assessed CaMKII phosphorylation of RYRs at serine 2814 after pro-arrhythmic pacing of LMS. After cryoinjury, increased CaMKII phosphorylation of RYRs was found in ENDO LMS, while CaMKII phosphorylation of RYRs was not changed in EPI LMS (Fig. 5 D).

These data indicate that inhibition of CaMKII reduces arrhythmogenic Ca^2+^ events that may induce ectopic beats. While reducing arrhythmic activity in ENDO after cryoinjury, we next assessed how CaMKII inhibition affects global cardiac function.

**Figure 6.**
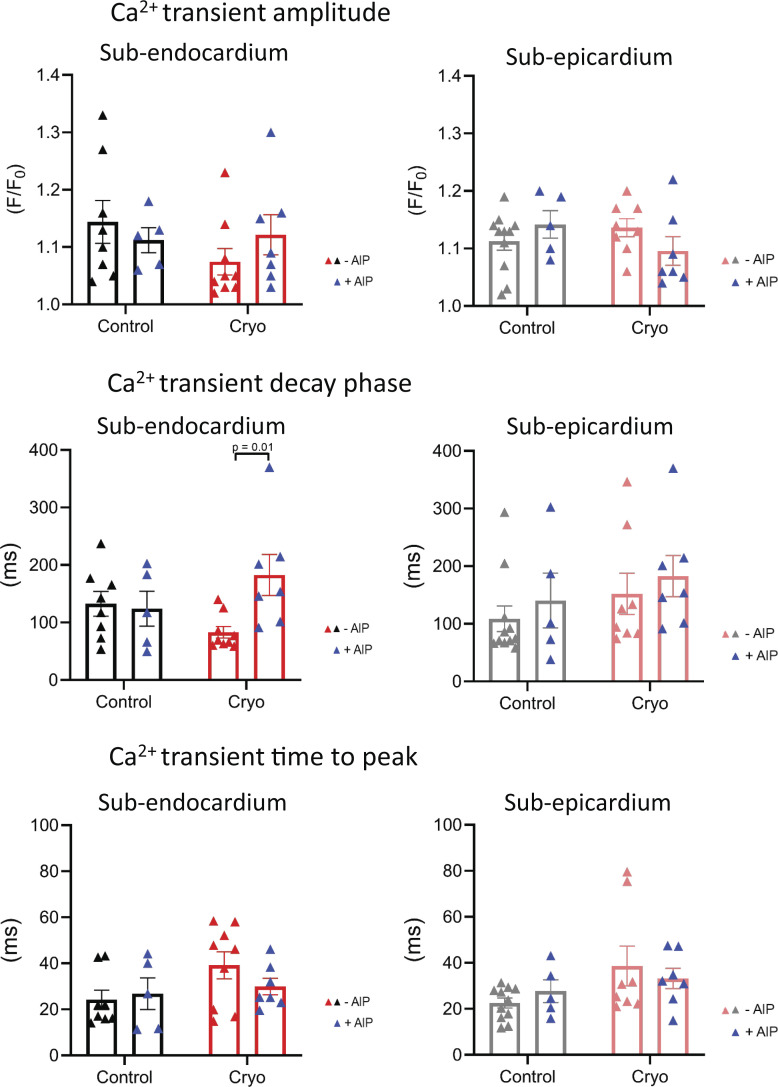
**CaMKII inhibition does not affect Ca^2+^ transient kinetics.** Mean values of Ca^2+^ transient kinetics during pro-arrhythmic pacing protocols in LMS from the ENDO in the absence (*n*_control_ = 8 from 8 rats; *n*_cryoinjury_ = 9 from 9 rats) and presence of AIP (*n*_control_ = 5 from 5 rats; *n*_cryoinjury_ = 7 from 7 rats; comparison with two-way ANOVA with Bonferroni post-hoc testing), and from the EPI in the absence (*n*_control_ = 11 from 10 rats; *n*_cryoinjury_ = 8 from 8 rats) and presence of AIP (*n*_control_ = 5 from 5 rats; *n*_cryoinjury_ = 7 from 6 rats).

During pro-arrhythmic pacing, inhibition of CaMKII with AIP in cryoinjured ENDO LMS did not significantly affect the Ca^2+^ transient amplitude or time to peak, but significantly prolonged the decay phase (Figs. 6 and S2). Ca^2+^ transient kinetics were not affected by AIP in control ENDO LMS, control EPI LMS, or cryoinjured EPI LMS (Fig. 6). Furthermore, the overall active force development during 1-Hz pacing protocols was not affected in ENDO or EPI LMS after cryoinjury (Fig. S3).

## Discussion

The use of organotypic cardiac slices offers a new tool to study transmural differences in arrhythmia initiation. Using Ca^2+^ imaging at high resolution, we studied SCR events in healthy and injured LMS from ENDO and EPI regions. The ENDO region showed a more marked propensity for local pro-arrhythmic events after cryoinjury. Moreover, using specific CaMKII inhibition to modulate SCR events, we showed that the injured ENDO was responsive to treatment. These data highlight the importance of transmural differences in the pathophysiology of arrhythmias and the need for regional-specific targeting in cardiac therapies.

### Cryoinjury of LMS as an in vitro model of cardiac disease

We used an in vitro model of cryoinjury to study arrhythmogenic events in the acute phase after tissue remodeling, as seen early after myocardial infarction. Current strategies to study myocardial injury mainly involve animal models and preparations from severe heart failure (HF) patients undergoing cardiac transplantation; however, several disadvantages limit the use of each system. Anatomical variations can result in variability in injury size in animal models, whereas mixed underlying disease pathologies and different disease stages restrict the understanding of underlying disease mechanisms when studying myocardium from HF patients. In this respect, our model offers a simpler and more reproducible approach that replicates the pathological stimuli in vitro using healthy LMS. Although the precise mechanism of inducing tissue damage with cryoinjury does not exist in human heart disease, the induced cellular remodeling, such as cell death and scar formation, are clinically relevant (van den Bos et al., 2005). In addition, our model shows local structural and functional remodeling of the surviving myocardium at the border zone of the cryoinjury, which is of clinical relevance, as recently shown in a large animal model of myocardial infarction (Dries et al., 2020). Loss of TATS and reduced Ca^2+^ transient kinetics in our cryoinjured LMS are in line with disease remodeling seen in animal models and human cardiac pathology (Dries et al., 2018; Heinzel et al., 2008; Louch et al., 2006; Lyon et al., 2009).

### Cardiac injury triggers more SCR events in the ENDO

Our data show more triggered events in injured ENDO LMS. Similar observations in the ENDO were found by Laurita and Katra (2005) using healthy canine tissue wedges with increased Ca^2+^ load and low-resolution optical mapping. Intrinsic differences in Ca^2+^ handling throughout the LV wall have been associated with these arrhythmic events in ENDO (Laurita et al., 2003; Laurita and Katra, 2005). Transmural gradients in APD may further modulate the electrical stability in different LV regions (Himmel et al., 1999). Advances in imaging techniques allowed us to record single SCR events in individual myocytes within a syncytium. Real-time tracking of single SCR events within the tissue showed that the distance of SCR propagation is within the limits of single cardiac myocyte dimensions (<100 µm). Moreover, SCR events propagated longitudinally along the direction of muscle fibers without having a transverse/lateral propagation. In line with previous reports, these data suggest that SCRs happen as intracellular rather than intercellular events (Lamont et al., 1998; Wasserstrom et al., 2010). The distance of propagation and velocity of Ca^2+^ waves are determined by the Ca^2+^ loading state of SR (Lipp and Niggli, 1994; Maxwell and Blatter, 2012; Wier et al., 1997). Ca^2+^ transients in the injured ENDO showed a faster decay phase, reflecting increased SR Ca^2+^ filling. In line with this, an increased local Ca^2+^ uptake by SERCA into the SR may facilitate Ca^2+^ wave propagation via the luminal sensitization of RYRs (Keller et al., 2007; Maxwell and Blatter, 2012). Conversely, SERCA2a gene expression and SR load have been shown to be reduced in the ENDO of HF patients (Lou et al., 2011; Prestle et al., 1999) and may differentially modulate SCR in the ENDO in a later stage of disease remodeling. In addition, an increased diastolic Ca^2+^ concentration also allows faster diffusion of Ca^2+^ by decreasing the cytosolic buffering capacity (Smith and Eisner, 2019), but this was not measured here.

After cardiac injury, SCR events occurred more and in a closer distribution pattern in ENDO LMS. A plausible explanation for these regional differences is the presence of transmural heterogeneity in the P_o_ of RYRs after cardiac injury. The P_o_ of RYRs is dependent on SR Ca^2+^ content, cytosolic Ca^2+^, and its regulatory proteins and RYR restitution (Bers et al., 1998; Shannon et al., 2002; Venetucci et al., 2007). Dysfunctional RYR and/or its regulatory proteins lower the SR Ca^2+^ content threshold to induce diastolic SR Ca^2+^ leak (Fischer et al., 2013; Venetucci et al., 2007). Posttranslational modifications of the RYR are known to modulate its gating and Ca^2+^ sensitivity (Niggli et al., 2013). Increased RYR phosphorylation at serine 2814 by CaMKII has been implicated in arrhythmogenesis in cardiac disease (Ai et al., 2005; Belevych et al., 2017; Dries et al., 2018; Fischer et al., 2014; Maier et al., 2003; Sag et al., 2009). Other modifications, such as phosphorylation by PKA and/or modulation by reactive oxygen species (ROS) or reactive nitrogen species, could also be attributed to an increased RYR P_o_ (Niggli et al., 2013). In addition, elevated SERCA activity in cryoinjured ENDO LMS accelerates local SR Ca^2+^ uptake. This increases the luminal sensitization of RYRs (Keller et al., 2007; Maxwell and Blatter, 2012) and allows the threshold for SR Ca^2+^ leak to be reached more quickly (Venetucci et al., 2007). Furthermore, increased expression of RYRs (Dilly et al., 2006) and/or shift in B1:B2 stoichiometry (Beau et al., 1993; Bristow et al., 1986) in the ENDO may also account for transmural differences. Further studies to address this point are warranted.

### Increased CaMKII activity in the ENDO

CaMKII has emerged as a potential major mechanism of pathological signaling in cardiac disease, such as HF, where its expression and activity have been shown to be upregulated (Ai et al., 2005; Hoch et al., 1999; Zhang et al., 2003). Our data show increased CaMKII-dependent phosphorylation of RYRs in cryoinjured ENDO LMS. In the ENDO, increased diastolic Ca^2+^ concentration and ROS have been reported (Andre et al., 2013; Laurita et al., 2003). Activation of CaMKII by Ca^2+^ and ROS is well known (Erickson, 2014) and may explain the increased CaMKII activation in ENDO LMS after cryoinjury. In line with our observations, Pan et al. (2018) reported increased CaMKII activation in the ENDO during high-frequency stimulation; however, it cannot be ruled out that increased Ca^2+^ concentrations and ROS directly modulate the P_o_ of RYRs (Niggli et al., 2013). In addition, other routes of CaMKII activation (i.e., nitric oxygen species, dO-GlcNAcylation; Erickson, 2014) or negative regulators of CaMKII activity (i.e., Kv4.3 subunit; Keskanokwong et al., 2011) may be different across the LV wall during cardiac disease.

Other proteins and sarcolemma ion channels may also be differently modulated by CaMKII in ENDO and EPI after cryoinjury. Phosphorylation of phospholamban increases SR Ca^2+^ uptake via SERCA (Mattiazzi and Kranias, 2014) and accounts for the fast decay phase in cryoinjured ENDO LMS. The modulation of Na_v1.5_ by CaMKII increases the intracellular Na^+^ concentration (Takla et al., 2020). High intracellular Na^+^ concentrations can prolong APD (Wagner et al., 2006), thereby increasing the susceptibility of myocytes to triggers, such as early afterdepolarizations (Hashambhoy et al., 2011), and elevate SR Ca^2+^ load via reverse NCX mode, increasing diastolic SR Ca^2+^ leak and DADs (Toischer et al., 2013). In contrast, changing the gating kinetics of Na_v1.5_ may predispose the myocardium to conduction block. The coincidence of conduction block with transmural dispersion of APD can precipitate reentry circuits (Cabo and Boyden, 2003). Furthermore, CaMKII also regulates the expression and channel gating of I_to_ (Mustroph et al., 2014) that may modulate APD dispersion and arrhythmia susceptibility differently throughout the LV wall. Moreover, intrinsic transmural APD dispersion favors electrical stability in the EPI and may be enhanced by an increased interaction of Kv4.3 and CaMKII in the EPI (Keskanokwong et al., 2011). More research is needed to investigate whether these other targets have a distinct modulation by CaMKII in different regions of the LV wall.

### Transmural differences in triggering ectopic beats in the injured LV

Our data show an increased potential of cryoinjured ENDO LMS to induce ectopic beats; however, the key question remains how SCR events can induce triggered activities in cardiac tissue, where electrical coupling of myocytes acts as a sink for depolarizing currents (Xie et al., 2010). To overcome this source-sink mismatch, modeling studies have suggested that a large number of myocytes (1,000–7,000,000 myocytes) must synchronously release Ca^2+^ in order for the aggregate to induce an ectopic beat (Chen et al., 2012; Xie et al., 2010). A more recent study suggested that a smaller number of cells (500–1,000 myocytes) is sufficient to induce an ectopic beat in rat trabeculae (Slabaugh et al., 2018). Moreover, the authors postulated that the typical endocardial surface with differently aligned fibers may decrease the electrical sink and thus promote triggering of ectopic beats originating from the ENDO (Slabaugh et al., 2018). Pathological conditions that reduce repolarization reserve or reduce electrical coupling, such as during fibrosis, can further decrease electrical sinks in HF hearts (Lang et al., 2017). Although early observations in our model did not show any differences in Cx43 cluster density or levels of collagen (data not shown), these mechanisms may further affect arrhythmia initiation at a later stage of remodeling. In addition, the structural penetration of Purkinje fibers in the ENDO can further increase the regional arrhythmia potential. Various studies have implicated spontaneous depolarizations and automaticity from Purkinje fibers, as well as Purkinje fiber-myocyte interactions that can modulate myocyte function (Bogun et al., 2006; Xing and Martins, 2004).

Synchronization of SCR is of paramount importance to trigger ectopic beats. Synchronization of SCR can either be achieved by direct cell–cell coupling (i.e., Ca^2+^ diffusion) or indirectly by synchronizing first latency timings (Izu et al., 2013). Given the absence of the intercellular diffusion of SCR, direct cell–cell coupling is unlikely to cause SCR synchronization. More likely is the impact of the first latency distribution of SCR events. If SCR event timing is widely distributed, SCRs will occur in some cells, but not in neighboring cells. Conversely, if SCR event timing distribution is narrow, then SCRs occur simultaneously in many cells and yield a higher overall inward current to depolarize the tissue and induce an ectopic beat. Our data showed a narrower distribution in first latency timings in injured ENDO LMS, highlighting the increased synchronization of SCR and its increased potential to induce ectopic beats. Additionally, the width of this latency distribution curve is highly dependent on SR Ca^2+^ load (Wasserstrom et al., 2010) and RYR restitution (Satoh et al., 1997). We did not measure SR Ca^2+^ content in the present study, but others reported reduced SERCA2a gene expression and SR load in the ENDO of HF patients (Lou et al., 2011; Prestle et al., 1999). This may implicate the presence of a bigger role in transmural differences in recovery from RYR refractoriness.

### Clinical relevance—potential for localized CaMKII treatment?

CaMKII has been implicated as an important therapeutic target in treating Ca^2+^-induced arrhythmias (Mustroph et al., 2017). Many reports have provided evidence that CaMKII overactivity can induce potentially lethal ventricular arrhythmias by initiating DADs and causing pro-arrhythmic tissue remodeling that favors reentry (Aistrup et al., 2017; Curran et al., 2010; van Oort et al., 2010; Yoo et al., 2018). Experimental use of CaMKII inhibition using small molecules and genetic approaches in animal models have been effective in preventing or reducing ventricular arrhythmias and pro-arrhythmic SR Ca^2+^ leak (Bezzerides et al., 2019; Chelu et al., 2009; Lebek et al., 2018; Neef et al., 2018). However, to date, the use of these inhibitors in vivo has been limited due to drug administration difficulties and off-target effects (i.e., neuronal versus cardiac CaMKII; Pellicena and Schulman, 2014). Our data add novelty to this concept and may steer current therapeutic strategies toward more localized and regional inhibition of this pro-arrhythmic kinase. In line with our data, recent evidence showed increased CaMKII activity in the ENDO compared with the EPI during high-frequency stimulation (Pan et al., 2018). Moreover, our results show that inhibition of CaMKII reduces SCR and ectopic beats in cryoinjured ENDO LMS without adverse effects on the ECC. Others previously reported similar observations of CaMKII lacking modulatory effects on the Ca^2+^ transient amplitude (Bezzerides et al., 2019; Daniels et al., 2018; Dries et al., 2018; Kohlhaas et al., 2006; van Oort et al., 2010). While inhibition of CaMKII is able to rescue hyperactive RYRs, other and/or additional signaling pathways may be involved in the modulation of ECC (Venetucci et al., 2013). Localized gene therapy in the ENDO may provide great promise (Katz et al., 2017, 2012) by using viral vectors that express CaMKII inhibitors under cardiac-selective promoters. Gene therapy using CaMKII inhibitors has recently been applied successfully in a mouse model of catecholaminergic polymorphic ventricular tachycardia (Bezzerides et al., 2019). Besides reducing regional triggered activities, regional inhibition of CaMKII may also reduce early afterdepolarizations by inhibiting reactivation of the L-type Ca^2+^ current in the ENDO, where I_to_ expression is low, and/or inhibit reentrant arrhythmias by reducing transmural APD dispersion gradients between epi- and endocardial myocytes. However, the involvement of these proposed mechanisms is beyond the scope of this study and requires further assessment.

### Limitations of the study

It is important to note that while our model describes observations in the rat myocardium, differences in Ca^2+^ handling throughout the LV wall have been observed in multiple mammalian species during various experimental protocols (Bondarenko and Rasmusson, 2010; Dilly et al., 2006; Laurita et al., 2003; Xiong et al., 2005); therefore, caution is needed when comparing these findings with other mammalian species. Moreover, the overall active force development of cryoinjured LMS was not affected, and we did not observe increased collagen deposits in cryoinjured LMS (data not shown). Therefore, this model should only be used to study acute remodeling after injury and/or for the assessment of therapeutic interventions acutely after infarction. Furthermore, the current experimental setup did not allow us to capture focal trigger points of ectopic beat initiation. While enlarging the field of imaging can increase the chance of capturing focal trigger points, it reduces the resolution of imaging for which this study was designed.

### Conclusions

In conclusion, we demonstrate that there is a higher susceptibility to pro-arrhythmic activity in the injured ENDO that is sensitive to CaMKII inhibition. Our data support the need for regional-specific targeting of CaMKII in the acute phase after cardiac injury. Furthermore, we show that the LMS technique represents a useful tool for studies of transmural heterogeneity upon myocardial remodeling.
